# Collectanea Medica, Consisting of Anecdotes, Facts, Extracts, Illustrations, Queries, Suggestions, &c.

**Published:** 1815-12

**Authors:** 


### COLLECTANEA MEDICA,

CONSISTING OF

### ANECDOTES, FACTS, EXTRACTS, ILLUSTRATIONS, QUERIES, SUGGESTIONS, &c.

RELATING TO THE

***History or the Art of Medicine, and the Auxiliary Sciences.***

***Memoir on the State and Composition of the Atmosphere, considered as the Cause of the Hospital Gangrene, or Putrescence among the Wounded.*** By Professor S. J. BRUGMANS, of Leyden.

(*Continued from p.* 405.)

SECTION 29. As all these unfavourable circumstances rarely occur, excepting in hospitals, and even in the military hospitals of cold climates, where the inhabitants participate in our mode of living, the denomination of hospital gangrene is readily understood.

§ 30. Among the peculiarities of this malady, arising from the nature of its causes, we may reckon further, the different degrees of its violence, and also of its tendency to spread (§ 10. 3). Here every thing depends on the combination of those causes and circumstances which excite the disease.

For instance, let us suppose that the general susceptibility of a great number of wounded is augmented, as observed § 12; and that, at the same time, one or more of the circumstances just mentioned are present: in this state of things, the wounds and ulcers may still, for a certain time, preserve a healing aspect. But, if one of the causes alluded to in § 23 and 26 has produced hospital gangrene, or if the contagious matter be brought there (accidents which frequently happen unknown to the surgeon), then, in a few hours, the atmosphere may be so loaded with miasmata, that the smell is distinctly perceived. Is it miraculous that, under these circumstances, the patients should be affected with hospital gangrene? though the surgeon may be surprised, if he is unacquainted with the symptoms which precede the gangrene properly so called.

§ 31. I shall here conclude this chapter, in which I have endeavoured to shew, that the proximate cause of hospital gangrene is a contagious matter, which is disengaged from the animal body under the circumstances above mentioned, and is communicated to the atmosphere, giving it the power of producing hospital gangrene in wounds and ulcers, with all the phenomena and symptoms which I have mentioned in the first chapter.

In the next chapter, I shall endeavour to ascertain the physical and chemical properties of an atmosphere thus infected.

### Chapter III.—*Of the State and Composition of the Atmosphere immediately producing Hospital Gangrene.*

*Of the State of the Air in Hospitals in general.*—§ 32. The examination of the qualities of the atmosphere charged with the miasmata of the hospital gangrene requires previously that the more general state of the air in the wards of the hospital should be ascertained. For this examination, we ought to select wards constructed according to the rules of art, as well with respect to situation, as to external division. I propose, therefore, the walls to be coated with lime, on both sides, throughout the whole length of the apartment; a dry floor; the windows placed at a proper height, as to permit free access of air and light; the height of the ceiling at least 10 or 12 feet; the length indefinite; the breadth 14 feet for a single row of beds, and from 18 to 20 for two rows; that the beds are detached, and placed 2 feet or 2½ from each other, and raised on feet 14 or 16 inches from the floor, each containing a single patient only; lastly, that the ward is heated by one or more stoves, and that at one end their are air-holes of a foot square, which open and shut at pleasure.

Let us now suppose all these beds occupied with patients whose diseases produce no contagious exhalation, or peculiar smell, such as ulcers in suppuration, eruptive or putrid fevers, &c.

In fine, I suppose that there is no source of putrefaction or of putrid exhalation in the vicinity. All the experiments which I am about to detail were made with the air of similar wards, taken in various hospitals, at different seasons of the year, and under all kinds of circumstances.

§ 33. It must also be previously remarked, 1st, that, although in hospitals well managed it is proper to leave open, during the night, one or more of the above air-holes, to give a free vent to the emanations which keep next the floor, from their specific gravity, I, nevertheless, closed them at night, always when I wished to examine the air of the wards; 2d, that in order to proceed to this examination, I collected the air very early in the morning, when the patients were scarcely all awake, and long before the fire fire of the stoves, or the morning employments of the nurses, had moved, mixed, or renewed the atmosphere.

§ 34. On entering a ward under these circumstances, an unpleasant smell, and a painful sensation in breathing, is felt. This is called by the Dutch *sapluet* (frousy). This air, however, does not emit any peculiar offensive smell. To conclude, the mass of air differs in its nature, at different altitudes of the room.

The mean stratum of this air most proper for respiration is that which is found at three, six, or seven feet from the floor.

According to my experiments, the mean proportion of the constituent parts of the atmosphere in general are as follow:*

| | | | | |
|---|---|---|---|---|
| Oxygen gas | 22 | 20 | 17 | 14 |
| Azotic ditto | 77 | 77 | 85 | 81 |
| Carbonic acid ditto | 1 | 3 | 4, 5 | 5 |

By comparing the extremes, we here see what has been frequently observed by chemists, viz. that, although the composition of the atmospheric air is changed by respiration and by emanations, this change, however, is less considerable than what we should at first suppose. Nevertheless, it is evident that the proportion of oxygen diminishes, while those of azote and carbonic acid increase, or are susceptible of increase.

§ 35. On examining the air above the mean stratum, I found that in the upper part the relative proportion of azote to the oxygen increases considerably, and that of the carbonic acid diminishes; and in the lower part the contrary takes place.

§ 36. At the height of two feet, and even sometimes of two feet and a half, the proportion of carbonic acid is generally from 8 to 12 per cent.; and, near the floor, 20 per cent. and upwards.

The flame of a candle nearly goes out close to the floor, and lime-water in an open vessel soon becomes turbid and milky.

§ 37. When, during the night, a vent has been given to the heaviest air by the opening of an air-hole close to the floor, the carbonic acid gas, as fast as it is developed, issues from the apartment; and, when a similar vent is given in the upper part of the room, we may still preserve in such an apartment, occupied by a number of persons, and even of sick persons, the ordinary state of the atmosphere, in a word, that which is most wholesome to mankind.

§ 38. Besides the constituent parts above-mentioned, which we know by the endiometer, and by the contact of the air with lime-water, there is no doubt that there are other principles floating in the atmosphere, especially where men are crowded together. The hygrometer shews the increase of the aqueous parts; and, by the smell, we perceive the presence of those animal substances, which are constantly separated, and particularly during the night, from the surface of the human body, but which escape all chemical tests.

* I used the endiometers of Fontana and Guyton.

Of

*Of the Composition of the Atmosphere loaded with the Contagious Matter of Hospital Gangrene.*—§ 39. It is sufficiently demonstrated by the phenomena described in the foregoing chapters, that the contagious matter of this disease is mixed with the atmosphere, whenever there are one or more patients affected with hospital gangrene; that, among a great number of wounded, if one only be affected with this disease, an experienced surgeon, on entering the ward, will discover it by the characteristic smell alone.

In my experiments, I made use of air which had been breathed by patients attacked with hospital gangrene, and in which the contagious matter was evident from the smell alone.

§ 40. In the first place, I thought it right to examine the proportions of the three constituent principles of the atmosphere by means of the endiometer and of lime-water. With this view, and in order to form just comparisons, these experiments were made with the same care, and under the same circumstances, as those mentioned in sections 32, 33, and 34.

Those who recollect the observations of Jurine, Gattoni, Alibert, &c. know that endiometrical experiments here are not very conclusive; that they are not sufficient to demonstrate the properties, having an eye to the salubrity of the air; but that they merely denote the presence of oxygen gas, the proportion of which is not very variable. Thus, Gattoni observed that the endiometer established no difference between the unwholesome air of the fetid marshes around Fuentes, near the sluices of the Valteline, and that of Mont Leguone.

Guyton Morveau found as much oxygen in air infected by meat in a state of putrefaction, as in that of the open atmosphere.

§ 41. The result of my experiments shews that the air of an infected ward contains the same proportions of oxygen and azote with that of another apartment (§ 34): this is not the case with carbonic acid gas, for we have observed that ulcers attacked with hospital gangrene exhale this gas in greater quantity than other sores. This greater proportion of carbonic acid proceeds from the ulcers themselves: we have only to place near them a glass of lime-water, and soon the small pellicle of calcareous carbonate will be manifested. Nevertheless this carbonic acid is not peculiar to those ulcers; all ulcers of a malignant nature, and from which an ichorous matter flows, give out a considerable quantity of this gas, and even ulcers which secrete a laudable pus, give it out also, although less abundantly.

§ 42. The smell of the ulcers, which is spread throughout the whole of the air of the ward, confirms the opinion that there exists a real putrefaction, and gives reason to suppose a certain quantity of free ammonia in the atmosphere. To assure myself of this, I placed in the vicinity of the patients, and afterwards close to their exposed ulcers, paper stained with pernambuc and tincture of turnsole, reddened by carbonic acid. Afterwards I made the same experiments in an atmosphere in which the emanations from the pus of these ulcers were as it were condensed and accumulated. With this this view, I shut up for some time in a box a considerable quantity of lint recently impregnated with pus from these malignant ulcers: the smell of the air, when afterwards examined, was intolerable.

These experiments have shewn, that neither in one case nor the other, had the change of colour indicative of the presence of the volatile alkali taken place: hence I concluded that these ulcers do not give out that substance.

§ 43. On the other hand, I always met with sulphureted hydrogen in this contagious atmosphere; and the following are my proofs:

1. When we agitate the air of the rooms in which those patients are, with a weak solution of nitrate of silver, we obtain a black precipitate, which is a sulphureted oxide of silver.

2. The solutions of acetate of lead, and of carbonate of lead, are changed into brown and black in this air.

3. If we mix oxygenated muriatic gas with this air in the hydropneumatic apparatus, a yellowish cloud is developed, and some time afterwards a yellow and pure sulphur is precipitated on the surface of the water, and against the sides of the vessel.

4. If we agitate distilled water with the air in question, renewing the quantity several times, and afterwards mix with this water oxygenated muriatic acid gas, it speedily becomes milky, and then the sulphur is visibly precipitated.

I think that chemical experiments leave no doubt as to the existence of the sulphureted hydrogen gas in this impure air.

The sulphureted hydrogen gas is still more sensible when we examine the air of the box just mentioned, in which lint has been deposited impregnated with pus.

§ 44. Although this gas emanates in such abundance from those ulcers that the air of the wards colours and precipitates the solutions of nitrate of silver and acetate of lead, yet this phenomenon is not calculated of itself to shew the contagious nature of hospital gangrene, for it takes place in all malignant, phagedenic, or ichorous ulcers.

I detected, for the first time, a few years since, the presence of sulphureted hydrogen gas in an open cancer of the cheek. Since that period, I have also remarked it in other ulcers of bad character.

However the case may be, after having seen that all these fetid ulcers, some more than others, and particularly those attacked with hospital gangrene (§ 23–27), infect the atmosphere in such a manner that hospital gangrene may be produced, it seems evident to me that the existence of sulphureted hydrogen proved by the above experiments demonstrates that hospital gangrene may follow its development.

The sulphureted hydrogen is not the miasma; but these two substances originate together, and the noxious effects of the contagious matter may be considerably augmented by the presence of this gas.

§ 45. The following experiments will shew, that, besides the above above substances, there is also in this corrupted air another principle which is peculiar to it.

Shake in a flask hermetically sealed the infected air of an hospital with pure water, or rather make it pass fifty times and upwards through this water, and you will find that the smell certainly becomes weaker, but that nevertheless it is not wholly destroyed.

The infected air may be treated in the same way, with lime-water, and by a solution of acetate of lead diluted in distilled water. By the first process, all the carbonic acid gas ought to be separated; and by the second, the sulphureted hydrogen gas: nevertheless, the remainder of the air being examined, there can be no doubt that it still emits a smell.

The same experiments were tried with air in which the emanations of the pus were condensed (as in § 42). After washing it in pure water, lime-water, and acetate of lead, the residue was distinguished far more strikingly by the smell peculiar to these ulcers, than in the preceding trials.

I conclude from all this, as has been already said, that there exists in the atmosphere thus infected a peculiar principle, chemically united to the air, and which still remains to be discovered.

Analogous experiments have taught me that in an atmosphere corrupted by the emanations of cancerous or scorbutic ulcers, or such as was loaded with the putrefaction of pieces of meat so strong as to give it the insupportable smell of putrefaction itself; that in such an atmosphere, I say, an animal matter is held in solution, which, like the air infected with hospital gangrene, cannot be detached from it, whether we wash it in pure water, in lime water, or in a solution of acetate of lead.

§ 46. After having separated from the corrupted air the carbonic acid gas by means of lime water, and the sulphureted hydrogen gas by the acetate of lead, I could not push my inquiries further by any chemical re-agent.

When, after these separations, we agitate the residue of this yet fetid air with newly distilled water, the mixture acquires the same smell; but the re-agents remain equally impotent over this solution.

I treated in the same way the air in which the constituent principles of the infected atmosphere was considerably thickened, according to § 42, and, notwithstanding the bad smell (both of the residue of the air, and of the water impregnated with this air) was very strong, none of the known re-agents in use exhibited the slightest change.

It results that the animal principle mentioned in § 45 is not acted upon by our re-agents.

§ 47. It appeared of importance to examine afterwards, by some experiments, the property of the infected air in accelerating or retarding the putrefaction of animal substances. With this view, in the month of July, 1807, at the moderate heat of 72° of Fahrenheit, I exposed to the action of the various kinds of air afterwards mentioned, in sixteen cylindrical glasses, as many small pieces of beef, all of an equal form, and each weighing an ounce.

The

The flesh was suspended in the glasses: the air was properly secured in them by means of water.

No. 1 was filled with pure atmospheric air, taken in a large garden.

No. 2 contained air taken at six o'clock in the morning, in an hospital ward, where there were twenty-eight patients affected with hospital gangrene: this air was taken at four feet and a half above the floor.

No. 3. The same air, previously washed in lime-water.

No. 4. Ditto, treated at first by lime-water, and afterwards by a solution of acetate of lead.

No. 5 was filled with the air of No. 3, with which was mixed two ounces in measure of oxy-muriatic gas. The piece of meat was placed in this mixture after the disappearance of the slight shade (§ 43–3).

No. 6. Air of No. 3, treated by oxy-muriatic gas, like No. 5.

No. 7. Air of No. 4, treated by the oxy-muriatic gas, as in No. 5.

No. 8. An air in which the emanations were considerably condensed by infected pus. (§ 42.)

No. 9. Air of No. 8, but previously washed in lime-water.

No. 10. Air of No. 9, but washed previously in lime-water, and treated afterwards by a solution of acetate of lead.

No. 11. Air of No. 8, mixed with four ounces in volume of oxymuriatic gas. The meat was placed in it, after the disappearance or precipitation of the slight cloud.

No. 12. Air of No. 9, treated by oxy-muriatic gas as in No. 11.

No. 13. Air of No. 10, treated in a similar way by oxy-muriatic gas, as in No. 11.

No. 14. Atmospheric air, first infected by exhalations from putrid meat, and afterwards washed in pure water, lime-water, and finally in a solution of acetate of lead.

No. 15. Was filled with atmospheric air (No. 1.) with which was mixed so much sulphureted hydrogen gas, that it operated on the re-agents as in No. 2.

No. 16. Atmospheric air, mixed with so much sulphureted hydrogen gas, that the mixture acted on the re-agents (§ 43.) as in No. 8.

**Changes in Pieces of Meat exposed to different kinds of air, in a succession of hours, as indicated at the head of each column.**

| No. | 3 hours. | 6 hours. | 9 hours. | 12 hours. | 15 hours. | 22 hours. | 36 hours. | 48 hours. | 60 hours. | 72 hours. |
|---|---|---|---|---|---|---|---|---|---|---|
| 1 | | | | a little discoloured. | discoloured. | discoloured. | colour degenerated. | commencement of putrefaction. | putrid humour. | |
| 2 | | a little flabby. | almost discoloured. | colour degenerated. | commencement of putrefaction. | putrefaction. | putrefaction with moistness. | decomposition. | | |
| 3 | | | a little discoloured. | a little less than No. 2. | colour degenerated. | a little less than No. 2. | commencement of putrefaction. | putrefaction. | decomposition. | putrefaction. |
| 4 | | | nearly like No.3. | a little less than No. 3. | a little less than No. 3. | a little less than No. 3. | as No. 3. | commencement of putrefaction. | a little less than No. 3. | |
| 5 | | | colour diminishes slowly. | as before. | | | first trace of tendency to putrefaction. | as No. 4. | commencement of putrefaction. | putrefaction. |
| 6 | | | as No. 5. | as No. 5. | | | as No. 5. | as No. 5. | | |
| 7 | | | as No. 5. | as No. 5. | | | as No. 5. | as No. 5. | | |
| 8 | flabby. | alone degenerated. | humid on the surface. | complete putrefaction. | putrefaction with softness. | decomposition. | very strong decomposition, with most offensive smell. | | | |
| 9 | | flabby. | colour degenerated. | aspect putrid. | a little putrid. | putrefaction. | putrefaction, with moisture. | | | |
| 10 | | a little flabby. | flabby. | as No. 9. | a little putrid. | putrefaction. | a little less than No. 9. | | | |
| 11 | | | colour diminishes slowly. | as before. | | first trace of tendency to putrefac. | as before. | commencement of putrefaction. | putrefaction inwards. | as before. |
| 12 | | | as No. 11. | as No. 11. | | as No. 11. | as No. 11. | as No. 11. | as No. 11. | as No. 11. |
| 13 | | | as No. 11. | as No. 11. | | as No. 11. | as No. 11. | as No. 11. | as No. 11. | as No. 11. |
| 14 | | already a little changed. | | colour degenerated. | commencement of putrefaction. | a little putrid. | putrefaction more considerable. | decomposition. | as before. | |
| 15 | | a little flabby. | flabby. | colour degenerated. | colour very much degenerated. | a little putrid. | putrefaction. | | decomposition. | |
| 16 | flabby. | colour degenerated. | colour very much degenerated. | as before, with more moisture. | a little putrid. | putrefaction complete. | commencement of decomposition. | total decomposition. | | |

It will be seen from this table that the meat undergoes changes nearly uniform in the various kinds of air, and that the difference consists principally in this, that periods of change are succeeded with more or less rapidity, until complete putrefaction takes place.

In the first instance, the meat loses its fresh colour and becomes soft and flabby, it afterwards takes the colour of ashes with here and there a greenish streak, the red colour entirely disappears, the surface becomes humid in whole or in part, and from that instant we perceive its bad smell, if brought into the open air. This humidity increases, the fibres are detached, and a putrid liquor flows from the meat, and the solid substances are dissolved, i. e. the putrefaction has advanced to a high degree, and all its products are visible. The substance preserves its solidity more in the gases with which the oxy-muriatic gas is mixed, than in any other aeriform fluid.

In short, these experiments give the following results:—

1. Infected air collected in the ward of an hospital, favours the putrefaction, No. 2.

2. The more the emanations of the infected pus are accumulated in the surrounding air, the more is the putrefaction facilitated. Compare Nos. 2 and 8.

3. The disinfection by lime-water diminishes the power which favours putrefaction in Nos. 2 and 8, but does not take it away entirely, (Nos. 3 and 9.)

4. The disinfection by the acetate of lead diminishes this faculty still more, without however taking it away; and even the tendency to putrefaction remains still stronger than in the atmospheric air. Compare Nos. 4 and 10 with No. 1.

5. We may conclude from what has been said, that the sulphureted hydrogen gas, added to atmospheric air, favours putrefaction as established by Nos. 15 and 16.

6. By comparing the results 3, 4, and 5, and the experiments 15 and 16, we see, as has been observed (§ 45), that, besides the principles demonstrated by chemical means, there exists also in the corrupted air another matter, which singularly favours putrefaction.

7. For the same reason, we see, in experiment 14, that putrid meat communicates to the air a similar peculiar matter, which, from its nature, favours putrefaction.

8. The oxy-muriatic gas takes from the corrupted air, Nos. 2 and 8, every thing which favours putrefaction in the atmosphere. This air so purified resists putrefaction longer than common atmospheric air. Compare Nos. 5, 6, 7, 11, 12, and 13, with No. 1.

§ 48. The following experiment throws still more light on the animal substance mentioned in § 45 and 47.

Shake in a close phial recently distilled water with corrupted air from one of the above wards; after having treated the air with lime-water and a solution of acetate of lead, repeat the operation several times with the same water and fresh quantities of the same air. The water, in this way, acquires the smell of the air, although the re-agents discover nothing in it, (§ 46); but when we preserve this water in a bottle hermetically closed for some days, or even some weeks, according to the season of the year, a putrefaction takes place, which cannot take effect in pure water; the volatile alkali is developed, and the water is turbid with small viscous flakes; a proof that the air had communicated to this water an animal substance although very minute.

§ 49. The minute animal substance, on which the peculiar smell of the corrupt air depends, generally endowed with the septic faculty, and which, when mixed with the atmospheric air, may certainly occasion hospital gangrene, seems itself to be the miasma properly so called, or at least the last and most subtle vehicle of the contagious substance.

From the examination of the products of its putrefaction, this substance is very probably composed of azote, hydrogen, and carbon. Its properties must be determined by the mode of combination of its constituent principles, as in all animal substances, and not merely by the constituent principles as such. It is for this reason that the chemical re-agents have no effect on it. Besides, we cannot doubt that this subtle animal substance is absorbed by the atmospheric air; it is generally known that there is no animal fluid, such as the blood, milk, bile, &c. which cannot, in a small quantity, be combined chemically with the atmosphere. It is thus that all these fluids communicate to the air the smell which is peculiar to them. The matter set free from blood or from milk when heated, those emanations called *peculiar principles* by the ancient chemists, and known under the denomination of *spiritus rector*, *aroma*, &c. are nothing but blood and milk dissolved in minute particles in the atmospheric air.

*Conclusions from what has been stated in this chapter.*

§ 50. After all that has been accomplished concerning the question—" Are there physical and chemical methods for ascertaining what state and composition of the atmosphere proves the immediate cause of hospital gangrene?"—I think I am entitled to answer in the affirmative.

In this atmosphere, we discover,

1. By the eudiometer, the diminution of oxygen gas, and the increase of azotic gas.

2. By lime-water, we see the increase of carbonic acid gas.

3. The nitrate of silver, acetate of lead, and oxy-muriatic gas, exhibit sulphureted hydrogen gas.

4. The characteristic smell, and the experiments which we have mentioned, demonstrate the presence of an animal matter manifesting itself particularly in putrefaction, the development of which it powerfully favours.

§ 51. By the presence of these substances, we can easily explain, in the following way, all the phenomena exhibited in the infected air of hospitals.

§ 52. The vital powers are diminished by the subtraction of oxygen and the augmentation of azote. Now we have seen it generally admitted, that the diminution of the vital powers infinitely increases all the causes of the diseases which tend to gangrene. This change of composition in the atmosphere not only disposes the patients to hospital gangrene, but also gives a fresh intensity *(momentum)* to the miasma properly so called.

§ 53. The same thing happens by the increase of the carbonic acid, and the more so as this stimulus seems also very injurious to the particular state of ulcers.*

§ 54. The inspiration of sulphureted hydrogen gas, which accompanies the miasma, and is at the same time disengaged from it, contributes greatly, according to well known facts, to diminish the vital powers. Thus this gas co-operates powerfully with the predisposition for hospital gangrene; but this agent becomes still stronger by the action which it exercises in the development of putrefaction.

§ 55. In short, the subtle animal effluvium mentioned in § 45, 46, 47, 48, and 49, and which is always mixed with the infected atmosphere contains the miasma, properly so called, capable of immediately producing hospital gangrene in wounds and ulcers. Whether this principle is separated from ulcers affected with hospital gangrene, or from other fetid ulcers; whether it proceeds from animal exhalation in general, or finally from animal substances in a state of putrefaction only; in all cases, not only is it disposed from its nature to putrefaction, but it also favours it in every thing with which it comes in contact. And, without doubt, it is sufficient that a principle so deleterious should be inspired, or should exert its influence over the surface of the body, and a portion of an open ulcer, in order that the vital powers of a wounded person may be attacked, and the tendency to putrefaction, nay, I will add gangrene, with all the concomitants of our hospital disease, shall shew themselves. Besides this general property of causing putrefaction, there is certainly added to the contagious matters of ulcers, affected with hospital gangrene, a specific power, which, applied to a suitable part, there produces, like other purulent substances or any given virus, a pathological state which procreates a substance in every respect similar to that which has produced it.†

In the same way as the pus, or exhalations of the pus, in small-pox, and in syphilitic ulcers, &c. produce the small-pox and syphilitic ulcers, so precisely, in ulcers affected with hospital gangrene, the matter coming in contact with the bodies of the patients, must

* In this passage, we suspect the author must mean the inspiration of carbonic acid gas.—Edit.

† What a circumlocution in the three succeeding paragraphs our author might have escaped, had he adopted our English phrase of morbid poison.—Id.

give give rise to a substance and condition quite analogous. Every fetid ulcer, every animal substance in a state of putrefaction, may also produce the disease in question; but this happens in a particular manner from the emanations of ulcers attacked with the true hospital gangrene.

After having clearly demonstrated, if I am not mistaken, what is the composition of the atmosphere which immediately gives rise to hospital gangrene, and from which this composition flows, it still remains for me to enquire into the means which may be opposed with success to this composition of the atmosphere.

*(To be concluded in our next.)*

---

*Sketches of the Medical Schools of Paris;* by JOHN CROSS, Member of the College of Surgeons in London, Corresponding Member of the Société Médicale d'Emulation of Paris, and late Demonstrator of Anatomy in the University of Dublin.

MR. CROSS's Sketches of the Medical Schools at Paris form an interesting little performance, but hardly a subject of criticism; we have, therefore, selected a few passages for this part of our work, by which the reader, who has leisure, will see how far his curiosity may be gratified by perusing the whole. The following will give some idea of that celebrated establishment l'Hotel Dieu.

" L'Hotel Dieu and la Charité are the great general hospitals, and from their central situation, are the only ones, besides l'Hospice de Perfectionnement, that are much frequented by students, during the winter season.

" The patients at l'Hotel Dieu, vary from fifteen hundred to two thousand, and generally approach near to the latter number. Besides the wards for medical and surgical patients, there is a ward for the reception of women actually in labour, or suffering abortion. The medical patients are far the most numerous, and eight or nine physicians are attached to l'Hotel Dieu. An account of the surgical wards, which chiefly attracted my attention, will give some idea of the whole of this immense institution.

" There are only two surgeons appointed to l'Hotel Dieu; one takes care of all the male, the other of all the female patients, and they change their departments annually. Before I speak of the surgeons, I must mention *les eleves de l'Ecole Pratique,* who assist in performing the duties of the hospital. *Les eleves externes*, or dressers, are chosen annually by the surgeons and physicians, after undergoing verbal examinations, and writing answers to some proposed questions; these appointments as dressers, which are received without any expence, each student can hold for either one or two years. The number of dressers is not limited; when I was at l'Hotel Dieu, above an hundred were attached to it, and there is consequently so little for each to do, that it is not an appointment of much value, unless we consider that those who receive it are obliged to attend regularly, are students of *l'ecole pratique*, and can therefore become candidates for higher situations. *Les eleves in-*

*ternes*

*ternes*, of the Parisian hospitals, correspond to our house-surgeons; they are chosen from among those of the *les eleves externes*, who have held their situations two years, according to the proofs they give of their attainments. *Les eleves internes* are appointed to all the hospitals; the number of them to each hospital is limited, and at l'Hotel Dieu there are nearly twenty. They have their separate apartments in the hospital, are boarded in it, and have, besides board and lodging, an annual salary of about twenty guineas each. They may retain their situation for two years, and there is a *Salle de Garde*, at which they take their days of attendance in succession, to receive accidents, &c. In the absence of the surgeons and physicians of the hospital, *les eleves internes* have the immediate care of all the patients. It is these *eleves internes* and *externes* only, whose education is at all attended to, and who take any share in the practical duties of the hospital. All other students are merely spectators; and every one, without fee, ticket, or ceremony, gets admission to the wards of the hospital at all hours, whether to follow the visits of the physicians and surgeons, to attend casual operations, or to examine by himself the state of any patient.

"It is hardly possible to describe this part of the discipline of the French hospitals, without adverting to what occurs in some great hospitals in London. At l'Hotel Dieu, none of the practical situations for students can be purchased; they are given to those who shew proofs of having attended to their studies. *Les eleves externes*, who merely apply the simplest dressings, are not appointed until after having shewn in their examinations, that they have profited by the gratuitous occasions of witnessing hospital practice; and there is security for a long attendance at an hospital, before they can be chosen *eleves internes*. From the manner in which the civil hospitals in England are supported, no such system as this, perhaps, would be practicable; but an amelioration, in some respects, might be effected without difficulty. A young man, who has money enough, becomes a dresser without reference to his qualifications, and I have known a youth from the country, who knew nothing of his profession but what could be gained from the use of the pestle and mortar, and who never before entered the wards of an hospital, commence as a dresser to a great hospital in London, and on his accident-day, have the first arrangement of a fracture, the dressing of a burn, the examination of a strangulated hernia, or the first treatment of an injury of the head. Whatever patients were admitted on a certain day, came under his care, and I have even seen him daily poking a bougie into the stricture of an irritable and inflamed urethra. A dressership to a large hospital is an office of some importance, and no one should be appointed to it, without having previously attended some public school of anatomy, and witnessed for a time the practice of an hospital.

"There is but one immense ward at l'Hotel Dieu, for male surgical patients, and it contains about two hundred and twenty beds. The patients are arranged according to their complaints, fractures, diseases diseases of urinary organs, abscesses, ulcers, injuries of the head, those who have suffered or are about to suffer operations, &c. M. Pelletan had the care of this ward, during my attendance there. In the midst of severe winter, by candle-light, he commenced his visit every morning at seven o'clock, and his arrival was made known to the students by the ringing of a bell. It is true M. Pelletan gave as much attention, sometimes, to a sore finger, or a half-cured simple fracture, that merely required the application of a bandage, as to some important cases; but he did not run through the wards, as if he was thinking all the time of his patients who were expecting him abroad, and of whom he was expecting a good fee. Twice or thrice in a fortnight, during his visit, and at uncertain days, he called over a list of the dressers and internal pupils attached to his ward, to ascertain who were absent, that they might be reprimanded, or dismissed from their office, if absent beyond a certain number of times. He seldom completed his visit in less than two hours; and at nine he commenced his clinical lecture, after which he saw the out-patients, who were admitted to receive advice daily.

"The clinical lectures, delivered at several hospitals in Paris, constitute one great superiority which they have over those of London, considered as practical schools. The attention of students is directed by them to individual and important cases, to the reasons for the adoption of any particular treatment, and to all that is most deserving of notice in the practice of an hospital. Dessault began to give lectures of this kind at l'Hotel Dieu, before the establishment of *l'Ecole de Santé*, but it is only since that period that they have been given at the other hospitals. The *clinique externe* at l'Hotel Dieu, is now given alternately, by M. M. Pelletan and Depuytren, five mornings in the week during the whole year. These lectures are delivered in the operating room, and they are free to medical students of every class and every country. *Les eleves externes et internes* must attend to assist the professors, and give an account of the patients under their care; and students who wish to obtain a degree at *l'Ecole de Medicine,* must pay for their inscriptions to these lectures, whether they choose to attend them or not.

"M. Pelletan, who has all the merit that can arise from a laborious cultivation of his profession, and a zeal for its improvement, is arrived at an age, when few men retain that degree of activity in all the mental faculties, so requisite to maintain good order in an extensive assembly of students, and satisfy them with the clearness and correctness of his lessons. I was much pleased however with the first lecture I heard, from the conviction I felt of the great advantages that must result from the effectual adoption of such a plan in any medical or surgical school. A patient had died of hydrothorax, in whom there existed piles. The rectum was taken out, to shew the morbid state of it, and the nature and treatment of the disease discussed. Another patient had died with strangulated inguinal hernia. Half the pelvis had been cut out, including the morbid morbid parts, of which a clean dissection had been made by one of *les eleves internes*, the abdominal muscles dissected into their different layers, and held out by wires, the condition and situation of the intestine shewn, the relation of the epigastric artery, the sac, the stricture, &c. There was no written history of the cases kept, none produced by the students; the particulars which the professor could not recollect, were supplied by *les eleves externes* or *internes*, who were allowed to make their remarks, and offer their suggestions.

"There are about one hundred and twenty beds in the female surgical wards, which communicate so freely with each other, that in fact there is only one large ward for all of them. Dupuytren visits it every morning, but is not very precise, as to the hour he arrives, nor the time he spends there. He is, however, rigorously attentive to the feelings, and immediate comfort of every patient, as far as is consistent with the necessary surgical treatment; and his conduct, in this respect, is an example of all that the medical man should be desirous of following.

"From the free admission to l'Hotel Dieu, and the rage that obtains for pursuing surgical, rather than medical practice, the wards are commonly so crowded with students, during the visits of the surgeons, that he who is desirous of instruction, is constantly interrupted by those who wish to see all, and who profit by nothing. Operations are performed with very little ceremony or bustle, and often without removing the patient to the operating room; a plan very considerate to the feelings of the patient, though ill calculated to extend the benefit of the operation to students. It is no uncommon thing for the operation for hernia to be performed during the morning visit to the ward, without disturbing the patient, or removing him from his bed, if the accident be in a state to require it at that time. This was more than once done whilst I was there, but I never saw well the successive steps of the operation. The best cases I witnessed, were where the surgeon was called to operate for hernia in the evening, on which occasions, through the kindness of one of *les eleves internes*, I was always sent for.

"A woman was admitted into l'Hotel Dieu, at one o'clock, with strangulated crural hernia of the right side. Her age was near fourscore. The hernia had been strangulated nine days, from the beginning of which time she had experienced vomitings; but the vomiting had entirely ceased within the last twenty hours, unless she attempted to take something into the stomach. It was five o'clock when I saw her. The surgeon had been sent for as soon as the history of her case was learnt, and no means had been tried, since her admission, to reduce the hernia, or give relief. She had had no stool since the strangulation commenced. The tumor was less than a hen's egg, and gave little pain when compressed. The abdomen was considerably distended and tense; the pulse was about 80, and feeble on account of her age. Dupuytren arrived at six, and after a very slight examination he observed, that all was in

'le plus mauvais état,' but that the operation was the only thing. He concluded, in short, that mortification had taken place, and the operation been too long delayed; but he did not take into the account, either the pulse or the countenance of the patient, neither of which indicated it. He commenced the operation immediately, without disturbing the position of the patient, or trying to reduce the tumor. So few pupils were present, that there was hardly a sufficient number of candle-holders and assistants. Dupuytren began by pinching up the skin transversely, puncturing it with the bistoury, and cutting upwards. The first incision thus made, was enlarged at both ends, and another was made that crossed it in the middle, at right angles. The operation was pursued, by dissecting back the four angles of skin. He scratched a hole in the successive layers, covering the tumor, and enlarged it with the scissars, still cutting the parts in correspondence with the crucial incision of the integuments. He was very cautious in these steps of the operation, remarking to us as he went on, that from the time the strangulation had existed, there might be great adhesion of the intestine to the fore part of the sac. At length a small puncture was made into the cavity of the sac, as was shewn by the escape of half an ounce of fluid. The sac was then laid open, by cutting in four directions from this point, (still corresponding to the first crucial incision) and the intestine, very unexpectedly, presented a favourable appearance, being of a dull red colour. Blood oozed freely through a slight scratch that had been accidentally made upon its surface. Anterior to the intestine, there was a small portion of omentum, which adhered firmly to the sac. A part of the lower and upper portion of the intestine within the abdomen, was pulled down, 'to see if it was in a good state.' The operator then put his fore-finger as far as the stricture would allow, and, passing a probe-pointed bistoury beyond the end of his finger, he cut the stricture slightly in a direction 'upwards, and a little outwards.' The intestine was readily reduced, but the small piece of omentum was still left in the sac, to which it was adherent. No attention was paid to closing the wound afterwards, and although, from the great laxity and abundance of the skin, the cut edges almost fell into apposition, no care was taken to keep them so. A thin piece of linen, spread with cerate, and having numerous small holes cut in it, was placed upon the wound; a compress and roller completed the dressing. The patient was ordered a half-pint emollient injection every four hours, and a little wine and water at the same time, on account of her weakness and great age. Shortly after the first injection, there was a copious evacuation, and it was not necessary to repeat it.

"Although an erroneous prognostic was given, and too little attention paid to the state of the patient, before the operation was commenced. I never saw it performed with less hurry, nor with more speed. This case did as well as possible. It does not however shew sufficiently the readiness of some of the French surgeons to have early recourse to the operation.

"On

"On another occasion, I was called to the hospital at six in the evening. A woman about forty years old, who had borne several children, had been admitted four hours before. She had long laboured under an umbilical hernia, which was reducible, productive of little inconvenience, and not the cause of her present symptoms. She had also, for a long time, had crural hernia, which became irreducible two days before, since which she had been occasionally troubled with vomiting, and passed no fæces per anum. She had not been treated surgically by any one before she came to the hospital, to which she was able to walk by herself, although she lived at the distance of a mile. During the four hours she had been in the hospital before my arrival, little or nothing had been done; the house-surgeon had put her into a warm bath for ten minutes, and tried feebly the taxis without success. I found a tumour in the left groin, big as a hen's egg; it resisted the slight attempts which I made to reduce it, but was very little painful when compressed, nor did it give the least mark of being actively inflamed. The patient complained of uneasiness in the umbilical region, more than of the tumor in the groin. She could pass no stool, experienced trifling anxiety, with a pulse scarcely quicker than natural, and not strong and full, so as to indicate, in any measure, the existence of much local inflammation, or the necessity for bleeding. I waited three hours. The patient had no vomiting whilst I was there, and I went away with a belief that, if the surgeon should come, he would not immediately perform the operation. When I returned to the hospital, the operation had been performed, and the intestine, which was found healthy, had been readily reduced. Several stools succeeded the exhibition of an enema, and the patient became quite easy. The dressings were not removed until the fifth morning after the operation. The wound, which had been prevented from healing by the first intention, was then about the size of a dollar, and nearly filled up to a level with the skin by granulations. Linen spread with cerate, and having numerous holes cut in it, to allow the discharge to pass through, was put to the sore, as at the first dressing; then plenty of charpie, compresses and roller. The woman remained about five weeks in the hospital, before the wound was entirely healed.

"In all the cases of hernia that I saw or heard of at l'Hotel Dieu, during my attendance there, the operation was had recourse to, as soon as the surgeon arrived. Where the pulse was strong and the patient in great pain, bleeding was employed; where strangulation had existed long, no means were tried. All attempts at reduction were made by the house pupils, and the surgeon was merely called to perform the operation. As far as regards the early performance of the operation, therefore, the French may be considered as excellent practitioners; and I believe, that in the practice of the hospitals, you will not be likely to find a patient's stomach loaded with purges, and the operation delayed by the surgeon, till it is impossible that its performance should be attended with success. Strangulated hernia may as justly be divided into acute and chronic, as any diseases, in which the danger and difficulty are to be estimated from the slow or quick progress of local inflammation. Although it be true, that it is better to perform the operation too soon than too late, the surgeon who has recourse to it indiscriminately at an early period, has some reason to be dissatisfied with his own conduct. Medical and surgical practice always tends to extremes, and the taxis is a very humble method of relieving the strictured intestine, compared with the operation.

"The examples I have given shew the general practice of the French surgeons, in the treatment of the wound, after operating for hernia; they hardly ever attempt to unite it by the first intention. A patient of Pelletan's had a large wound in the groin sixteen days after the operation, when it was attacked with hospital gangrene; carbon mixed with *eau-de-vie* was applied; the sloughing did not extend far; the patient was still in the hospital at the end of two months, with a large granulating wound. The intercourse between England and France has been so little, during above twenty years of almost uninterrupted war, that great obstacles have been thrown in the way of mutual improvement: yet it is difficult to conceive, why the good practice of the English surgeons, in the treatment of wounds after operations is not to be found in the French hospitals. To attempt immediate union after amputation, is a practice far from being universally followed in them, and within the last year a Memoir has been published, to prove the safety and advantage of sometimes making the attempt. The author states, that under the common treatment, where the wound is filled with charpie, the cure in the most favourable cases does not take place in less than ten weeks, and is often prolonged to three or four months; whilst in the few instances, in which he had tried a different method, he had sometimes healed the stump in less than three, and always within five weeks. M. Roux, however, adds 'he is far from thinking, that to try to effect immediate union, can be established as a general practice, or that it is applicable to all cases without distinction.'

"Previous to the Memoir of M. Roux, the practice it recommends had notwithstanding been adopted by some of the Parisian surgeons, and by the best surgeons in the army; and M. Percy, in his *rapport* on the above Memoir, mentions an extraordinary instance of success under this treatment. 'Ninety-two patients suffered amputation of the thigh, leg, or arm, by the same hand, on the 22d of September, in the affair of Nurembourg, and eighty-six of them were cured by the eighteenth of the following month.'

"The Parisian surgeons do not often succeed in their attempts to procure immediate union after amputation, which is to be attributed to other causes, besides the bad air of a large metropolis, or of a crowded hospital. Although we have reason, in most instances, to admire their dexterity in the mechanical parts of surgery, they are awkward in supplying the adhesive plaster, which is commonly very clumsily and thickly spread upon coarse linen. To employ means for carrying off heat, and for preventing its accumulation, is often often as necessary to insure immediate union, as to adjust the surfaces of the wound, or to apply accurately the adhesive plaster: consequently we may expect the result in cases where the stump is loaded with charpie, compresses and bandages, to be different from what happens in those where it is exposed to the air, with scarcely any covering besides the retentive strips of plaster.

"Improved methods of treatment ought, in a great measure, to be valued according to the facility of adopting them, and the frequency of the diseases they are intended to relieve. It is for these reasons, that the employment of the adhesive strap in ulcers of the legs, ranks deservedly high amongst the improvements in British surgery. Its employment, however, has been very slightly noticed by a French author, whose works have been much read in this country. 'Un chirurgien Anglais,' says M. Richerand, 'a employé avec succès les emplâtres agglutinatifs, dans la vue de ramener la peau sur la surface découverte par l'erosion ulcéreuse. J'ai fait usage du même moyen, et observé dans tous les cas qu'il avance évidemment de plusieurs jours l'entière cicatrisation. Mais cette termination de la maladie est-elle toujours desirable, et peut-on tenter sans danger la guérison de toutes sortes d'ulcères?'

"As I never saw cold applications employed in any cases of local inflammation, at the French hospitals, I may be allowed to say, that they are not much used. The Parisian surgeons, who have lately visited London, seem indeed to have carried back with them very little of what comparatively forms the best part of English surgery, the treatment of patients before and after operations, the cure of local diseases by constitutional means, the regulating of inflammation by diminishing the temperature of the part affected, and the use of sticking plaster for ulcers, recent wounds, and granulating sores.

"The hospital surgeons have reason to be doubly anxious to procure immediate union after operations, in order to diminish the chance of hospital gangrene attacking the wound. When the allies, a twelvemonth ago, were near Paris, the number of patients in l'Hotel Dieu was double what it is at present, and hospital gangrene was very frequent. The beds are still too numerous, nor do carefulness in ventilating the wards, or strict attention to cleanliness, entirely prevent the occurrence of this dreadful disease. I was told by several of the French surgeons, that they did not rely at all on internal means for stopping the progress of hospital gangrene, and that their experience had proved them to be insufficient, if not wholly inefficacious. Dupuytren, in reply to the account I gave him of the practice and opinions of English surgeons on this subject, assured me that he had no confidence but in local applications, and that internal remedies alone, as far as he had found, did almost nothing. The same remark has been made in a very recent publication on hospital gangrene, although it seems to be rather at variance with its being a constitutional and contagious disease, which the author has admitted. Experience, however, must be trusted to, and the French surgeons have had had abundant occasions of witnessing this disease, in their civil as well as military hospitals. Vegetable and diluted mineral acids are the local means employed with effect in mild cases. I have already alluded to a case of Pelletan's, where carbon was applied, and the progress of the disease impeded. But the actual cautery is the only mean that has been found effectual, in stopping the fatal progress of bad cases of hospital ulcer, and the iron is applied red hot, so as to produce an eschar on every point of the surface of the sore.

The application of the actual cautery gives insupportable torture, and there is something so apparently barbarous in the use of it, that we should hardly expect the surgeon wonld employ it, except where he hopes to save life, or to give great relief. It is however used by the French surgeons, in many cases, besides the hospital ulcer. An aged man, suffered from a cancer of the lip, which had proceeded so far as to affect the jaw bone. Several consultations were held to decide upon the operation, to which the patient at length submitted. The whole lower lip was removed, and there was nothing left to cover the lower jaw, and close the mouth. At the end of three weeks, granulations, of an unfavourable appearance, and furnishing a very fœtid discharge, had arisen from the cut surfaces, and covered the mental portion of the jaw. The *pommade d'arsenic* was proposed to be applied, but the following treatment was decided upon in preference. The students surrounded the patient, who was placed on a chair in the middle of the ward, and near him was a red hot cauldron, with irons of different forms, square, round, triangular, and diamond-shaped. M. Pelletan, after brandishing one of these fiery weapons in the air, applied it to the cancerous surface, and kept it there several seconds. He applied four or five of these red hot irons, of different forms, till the whole surface was an eschar. Two days afterwards, the eschar had separated; the whole mental portion of the lower jaw was bare, the mental foramen on one side was exposed, and at the centre of the chin the bone was black, where the actual cautery had reached, and destroyed it. I did not remain long enough at l'Hotel Dieu, to witness either the exfoliation of the dead bone, or the death of the patient.

"In one case, I thought the actual cautery was used with good effect. A middle aged woman had a very large tumor of the cheek, formed by a fungus, which originated in the right antrum highmorianum. A part of the malar and superior maxillary bones had been destroyed by the progress of the tumor, but the skin of the cheek remained entire. The tumor was removed through the mouth, by separating the cheek from it on the inside, and a portion of the diseased malar and maxillary bones taken away. The parts of the tumor most deeply situated, and which could not be removed by the knife, were destroyed by the application of four or five red hot irons. Inflammation of the eye, and slight eversion of the lower lid, were the only inconveniencies after the operation, and at the expiration of two months, the patient was perfectly well, well, with little more deformity than a flatness of the cheek. Three or four fingers could be passed into the immense cavity within the cheek, even to the bony floor of the orbit."

"Before the French revolution, l'Hotel Dieu was constantly crowded with above four thousand patients. There were few small beds for containing a single patient. Most of the beds were large enough to hold four or even six persons; and whether in the wards for surgical cases, for fevers, for contagious diseases, for *femmes accouchées,* &c. four or five patients were commonly put into one bed. How can we wonder that about the same period, the two most eminent surgeons in France and England should pursue diametrically opposite modes of practice? that whilst Pott spoke of trepanning as an operation of little danger, and to be performed in all cases of fracture, Dessault almost proscribed its performance in any case?"

"I have already described enough to shew that l'Hotel Dieu deserves no longer to be called a "storehouse of corruption and disease," language which has been unjustly applied to it of late years. A revolution has taken place in the internal administration as well as in the surgical practice of this institution. The practice of Dessault, with respect to trepanning, is not at all followed at l'Hotel Dieu, nor any other hospital."

The following is from the chapter on l'Hospice de la Maternité.

"Epidemic diseases commonly find their way into large hospitals. Puerperal fever has been very destructive both at l'Hospice de la Maternité and the Lying-in Hospital of Dublin. An immense number of children at la Maternité have died from a disease that rarely occurs any where else than in hospitals for infants, *l'endurcissement du tissu cellulaire.* Not less than one in twenty have been attacked with this disease. It most often attacks the extremities; frequently the neck, face, or abdomen. The parts affected become swollen, hard, cold to the touch, and of a dull red or livid colour, not pitting from nor yielding to pressure with the finger. When the lower extremities are affected, the soles of the feet become convex. On dissection, the lymphatic and generally the mesenteric glands are found much enlarged, and the cellular substance is distended with a yellow serous fluid that is coagulable by boiling water. This disease, when it affects the cheeks, has many symptoms in common with trismus; but I cannot agree to the remark Dr. Jos. Frank made, at the Foundling Hospital of Paris, that it has any very great resemblance to tetanus. The appearance, the attitude of the body, the sensations given by the surface of the body to the touch, are different. The appearances on dissection are very different, for in tetanus no information is gained by dissection, any thing unnatural being rarely or never observable. Trismus nascentium attacks a great number of children shortly after birth in the Dublin Lying-in Hospital, and although I have been favoured with opportunities of dissecting them where this disease had proved fatal, I never could find any morbid appearances in the throat, viscera of the cranium, thorax, abdomen, or any other parts of the body."

CRITICAL

